# EBV-Driven Lymphoproliferative Disorders and Lymphomas of the Gastrointestinal Tract: A Spectrum of Entities with a Common Denominator (Part 2)

**DOI:** 10.3390/cancers13184527

**Published:** 2021-09-08

**Authors:** Magda Zanelli, Francesca Sanguedolce, Andrea Palicelli, Maurizio Zizzo, Giovanni Martino, Cecilia Caprera, Valentina Fragliasso, Alessandra Soriano, Luca Valle, Stefano Ricci, Fabrizio Gozzi, Luca Cimino, Alberto Cavazza, Francesco Merli, Stefano A. Pileri, Stefano Ascani

**Affiliations:** 1 Pathology Unit, Azienda USL-IRCCS di Reggio Emilia, 42123 Reggio Emilia, Italy; andrea.palicelli@ausl.re.it (A.P.); stefano.ricci@ausl.re.it (S.R.); alberto.cavazza@ausl.re.it (A.C.); 2 Pathology Unit, Policlinico Riuniti, University of Foggia, 71122 Foggia, Italy; francesca.sanguedolce@unifg.it; 3 Surgical Oncology Unit, Azienda USL-IRCCS di Reggio Emilia, 42123 Reggio Emilia, Italy; maurizio.zizzo@ausl.re.it; 4 Pathology Unit, Azienda Ospedaliera Santa Maria di Terni, University of Perugia, 05100 Terni, Italy; gio.martino@gmail.com (G.M.); ceciliacaprera@libero.it (C.C.); s.ascani@aospterni.it (S.A.); 5 Laboratory of Translational Research, Azienda USL-IRCCS di Reggio Emilia, 42123 Reggio Emilia, Italy; valentina.fragliasso@ausl.re.it; 6 Department of Pathology, Case Western Reserve University, Cleveland, OH 44106, USA; alessandra.soriano@ausl.re.it; 7 Gastroenterology Division, Azienda USL-IRCCS di Reggio Emilia, 42123 Reggio Emilia, Italy; 8 Anatomic Pathology, Department of Integrated Surgical and Diagnostic Sciences (DISC), University of Genoa and Ospedale Policlinico San Martino, 16132 Genoa, Italy; valleluca9@gmail.com; 9 Ocular Immunology Unit, Azienda USL-IRCCS di Reggio Emilia, 42123 Reggio Emilia, Italy; fabrizio.gozzi@ausl.re.it (F.G.); luca.cimino@ausl.re.it (L.C.); 10 Hematology Unit, Azienda USL-IRCCS di Reggio Emilia, 42123 Reggio Emilia, Italy; francesco.merli@ausl.re.it; 11 Haematopathology Division, European Institute of Oncology-IEO IRCCS Milan, 20141 Milan, Italy; stefano.pileri@unibo.it

**Keywords:** Epstein–Barr virus, plasmablastic lymphoma, primary effusion lymphoma, Burkitt lymphoma

## Abstract

**Simple Summary:**

Epstein–Barr virus (EBV)-associated lymphoproliferative disorders have been garnering attention in recent years. EBV infects people primarily in their first years of life. The viral genome is maintained in a latent phase within the host cells, usually B-lymphocytes. The immunosuppression of different origins alters homeostasis between the virus and the host, enabling EBV-linked lymphoproliferative disorders to appear. Entities with different biological behaviors often share some morphological and phenotypic features, making the diagnosis complicated. Nodal and extra-nodal sites, including the gastrointestinal tract may be involved. This review, divided into three parts, aims to summarize the available clinical, pathological, molecular and therapeutic data on EBV-associated lymphoproliferative disorders involving the gastrointestinal tract. In this part of the review, we discuss plasmablastic lymphoma, extra-cavitary primary effusion lymphoma and Burkitt lymphoma.

**Abstract:**

Epstein–Barr virus (EBV) is a common pathogen infecting people primarily early in life. The virus has the ability to persist throughout a person’s life, usually in B lymphocytes. Conditions of immunodeficiency as well as the introduction of immunosuppressive therapies and the advent of transplant technologies has brought immunodeficiency-associated lymphoproliferative disorders into view, which are often driven by EBV. The group of EBV-associated lymphoproliferative disorders includes different entities, with distinct biological features, ranging from indolent disorders, which may even spontaneously regress, to aggressive lymphomas requiring prompt and adequate treatment. These disorders are often diagnostically challenging due to their overlapping morphology and immunophenotype. Both nodal and extra-nodal sites, including the gastrointestinal tract, may be involved. This review, divided in three parts, summarizes the clinical, pathological, molecular features and treatment strategies of EBV-related lymphoproliferative disorders occurring in the gastrointestinal tract and critically analyzes the major issues in the differential diagnosis. In this part of the review, we discuss plasmablastic lymphoma, extra-cavitary primary effusion lymphoma and Burkitt lymphoma.

## 1. Introduction

EBV represents the most common persistent virus in humans, as after primary infection in the first years of life, the virus has the ability to latently persist in B lymphoid cells [1,2,3,4,5,6,7,8,9,10,11,12]. Since the discovery of the virus more than 50 years ago, EBV contribution to the development of a wide and expanding spectrum of lymphoproliferative disorders (LPDs) has been recognized.

While EBV is the common denominator of entities with different pathological features and clinical behaviors, other factors, including the state of the host immune system, iatrogenic immunosuppression (IS), age-related immune-senescence and genetic alterations, play important roles in the pathogenesis of EBV-associated LPDs [1,2,3,4,5,6,7,8,9,10,11,12].

The incidence of different entities varies according to the geographical areas.

Some EBV-positive LPDs, such as EBV-positive diffuse large B-cell lymphoma, not otherwise specified (EBV-positive DLBCL, NOS) and Burkitt lymphoma (BL), have a higher incidence in areas with high rates of EBV infection [8]. Depending on the infected cell type, EBV-positive LPDs are divided in B-, T- and NK-cell categories [8,9]. These disorders may occur in both nodal and extra-nodal sites [8,9].

For therapeutic reasons, the correct identification of different lymphoid entities linked to EBV is essential, and it may be diagnostically challenging as different entities share morphological and immunophenotypic features.

EBV is associated with many tumors, including epithelial, mesenchymal and hematopoietic neoplastic proliferations.

The EBV-associated lymphoid disorders are summarized in Supplementary Table S1.

The gastrointestinal tract (GIT) is a common site of EBV-driven LPDs.

In this review, we focus on LPDs that develop in the GIT, in which EBV detection is considered a diagnostic parameter. Some of these entities may even develop in inflammatory bowel disease (IBD). The mechanism underlying the association of IBD with EBV-associated hematopoietic disorders still remains not completely clarified [13,14,15,16,17,18,19,20,21,22,23,24,25,26]. The multi-agent immunomodulatory treatment of IBD and EBV infection, more than IBD itself, may have potential roles in LPDs, as mentioned in the first part of this review, which discussed EBV-positive mucocutaneous ulcer (EBVMCU) and classic Hodgkin lymphoma (cHL), which may occur in IBD patients [13,14,15,16,17,18,19,20,21,22,23,24,25,26,27,28,29].

Our review consists of three parts. In this part, we describe the clinicopathological features of plasmablastic lymphoma (PBL), extra-cavitary primary effusion lymphoma (EC-PEL) and Burkitt lymphoma (BL).

In part 1 of the review, we discussed EBVMCU, EBV-positive DLBCL, NOS and cHL [8].

In part 3, we will analyze, post-transplant lymphoproliferative disorders (PTLDs); chronic active EBV (CAEBV) infection of T- and NK-cell types; and extra-nodal NK/T-cell lymphoma (ENKTL), nasal type [8].

The exact role of EBV in the pathogenesis and development of many LPDs is often not completely elucidated.

Among the entities discussed in the present paper, BL represents the first example of a virus-associated lymphoma. Due to the different associations of EBV in the three variants of BL and the restrictive latency pattern with only the expression of EBNA-1, some authors suggest that the virus may not be the initiating event for BL, with the oncogenic capacity of EBNA1 being controversial [2,30]. Recently, the importance of a polymicrobial environment in the pathogenesis of BL has been hypothesized [31].

PEL is strictly linked to Kaposi Sarcoma Herpes Virus/Human Herpes Virus 8 (KSHV/HHV8), which is considered the main driving force in its pathogenesis; EBV is not present in all cases of PEL and it is supposed to play a cofactor role [32].

Similar to PEL, in PBL, EBV is identified in a proportion of cases and it is likely to have a pathogenetic role in conjunction with other factors such as *MYC* gene rearrangement [30]. Further studies, even with the use of new technologies such as next generation sequencing analysis, might help in better understanding the exact role of EBV in the pathogenesis of EBV-associated LPDs.

## 2. PBL

### 2.1. General Features and Etiology

PBL, initially considered a morphological variant of diffuse large B-cell lymphoma (DLBCL), was identified as a distinct entity starting in the 2008 WHO classification [33].

PBL is a high-grade lymphoma initially reported in the jaw and oral cavity of young, HIV-positive males [8,34,35].

It may develop even in the context of other causes of IS, including autoimmune disorders, post-transplant, iatrogenic IS and age-related immune-senescence [36,37]. Immunocompetent individuals may also be affected [36,37].

The disease shows a male prevalence of 4–5:1, occurring at any age, although HIV-positive individuals may develop PBL at a younger age (median 42 years) compared with PBL associated with other conditions of IS (median 60) [8,38].

PBL is predominantly an extra-nodal disease, with a predilection for the oral cavity in HIV-positive individuals, with the GIT being the second most common site. Other extra-nodal sites such as the skin, bone, genitourinary tract, sino-nasal tract, central nervous system (CNS), liver, lung and orbits are more rarely reported [8,34,35,36,37,38,39,40,41,42,43,44,45,46,47,48,49,50,51,52,53,54,55,56,57,58,59,60].

Nodal involvement is rare, particularly in HIV-positive cases, whereas it is more frequently observed in post-transplant cases [37].

EBV is likely to have a pathogenetic role in PBL occurrence. Particularly in the context of HIV infection, a large number of PBL cases harbor EBV as documented by EBV-encoded ribonucleic acid (EBER) in situ hybridization (ISH) [39]. However, other mechanisms must be involved as EBER is negative in approximately 30% of PBL cases. The mechanism of EBV-related lymphomagenesis consists of different steps, including the prevention of apoptosis induced by several viral-encoded products and the blockage of hypomethylation in B cells causing their immortalization [40].

### 2.2. PBL and GIT

The GIT represents the most common extraoral site, accounting for approximately 30% of extraoral PBL [38].

In GI PBL, male patients are affected almost twice as often as female [41] and the median age is around 50 years; similar to other localizations, HIV-positive patients are younger (approximately 40 years) than HIV-negative one (more than 60 years) [41].

The rectum and anal canal have been reported as the most commonly involved sites, particularly in HIV-positive patients [42,43]. In a recent review, the most common GI sites were the stomach; small bowel; and large bowel, including the colon and rectum, in decreasing order [39]. The possible reasons for these differences might be the prevalence of the HIV-negative cases evaluated and the high number of patients with Crohn’s disease, which is typically a disease of the small bowel [39].

The association between IBD and non-Hodgkin lymphomas, including PBL, is well-known. The risk of lymphoma in IBD patients seems to be related to the treatment more than to the disease itself, in particular with thiopurines and to a lesser extent with anti- tumor necrosis factor α (anti-TNFα) [39,44,45].

In GIT, PBL presents, commonly, as a mass and/or a polypoid lesion [42] and, more infrequently, as a small-sized colonic stricture [46] or as superficial involvement of the whole large bowel [47]. At diagnosis, most patients are at the advanced stage (III/IV). At presentation, bone marrow (BM) involvement is more common in HIV-positive patients (75% versus 50% in post-transplant and 25% in cases without known causes of IS) [8].

### 2.3. Histology, Immunophenotype and Genetic Profile

Plasmablasts (PBs) are large cells with eccentrically located large nuclei, evident nucleoli, basophilic or amphophilic cytoplasm, often with a perinuclear clear halo.

**Figure 1:**
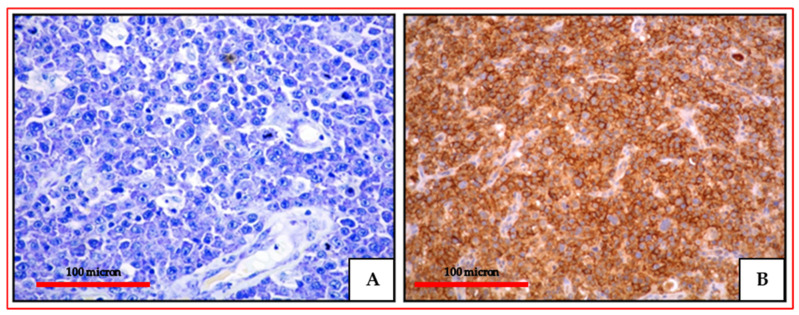
(**A**) PBL of the colon: high power view showing a diffuse proliferation of large-sized cells with evident nucleoli (Giemsa staining, magnification 400×; original image from Prof Ascani); (**B**) PBL of the colon: diffuse EMA expression of the neoplastic cells (immunostaining, Ventana immunostainer, magnification 400×; original image from Prof Ascani).

However, PBL shows a wide morphologic spectrum ranging from cohesive and monomorphic proliferation of large cells with immunoblastic, plasmablastic or anaplastic features to proliferation of cells with a clear-cut plasmacytic differentiation (Figure 1).

Geographic necrosis can be present, and a starry sky pattern with apoptotic bodies and tingible body macrophages is common.

**Figure 2:**
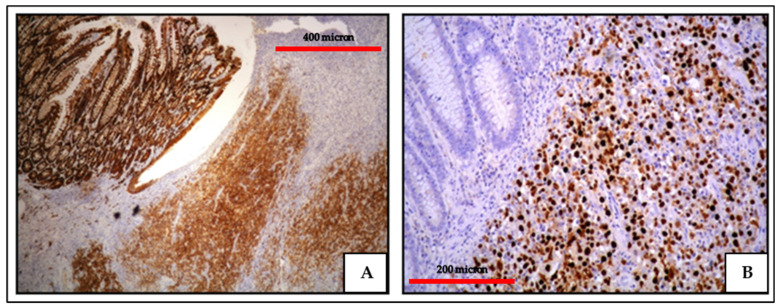
(**A**) PBL of the colon: MUM18 expression of the neoplastic proliferation (immunostaining, Ventana immunostainer, magnification 100×; original image from Prof Ascani); (**B**) PBL of the colon: diffuse EBV positivity of the neoplastic cells (EBER-ISH, magnification 200×; original image from Prof Ascani).

The neoplastic cells express a plasma cell phenotype (MUM1/IRF4, MUM18, CD138, VS38c, CD38 and PRDM1/BLIMP1) (Figure 2).

**Figure 3:**
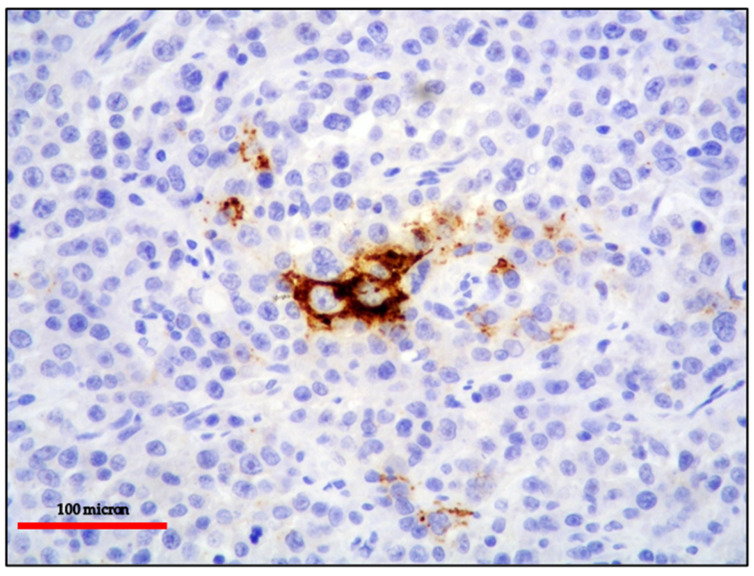
PBL of the colon: the lack of B-cell marker expression is a feature common among neoplasms with plasma cell differentiation such as PBL and PEL (CD20 immunostaining, Ventana immunostainer, magnification 400×; original image from Prof Ascani).

CD45 and B-cell markers (CD20 and PAX5) are generally negative (Figure 3) or weakly positive in a small number of cells; CD79 alpha is positive in approximately 40% of cases. EMA and CD30, as well as cytoplasmic immunoglobulins, most commonly IgG, are often expressed. BCL2 and BCL6 are negative whereas CD10 is positive in about 20% of cases [36]. The proliferative index (ki67) is elevated. HHV8 is negative. T-cell markers may be aberrantly expressed, more often in EBV-positive cases and in immunocompromised hosts [39]. CD56 may be found in 25% of cases, an overlapping feature with multiple myeloma (MM).

MYC protein is usually overexpressed and not limited to the *MYC*-rearranged cases, suggesting alternative mechanisms of *MYC* activation and protein expression. EBER is positive in 60–75% of cases, more frequently in HIV-positive and post-transplant patients. In EBER-positive cases, LMP1 is rarely expressed, indicating type I latency pattern. The latency pattern type II is observed in PBL arising in HIV-positive individuals or in transplantation [39]. EBER-positive PBLs are usually associated with the expression of programmed cell death protein 1 (PD-1/PD-L1), *MYC* rearrangement and a better outcome than EBER-negative PBL [58,59,60].

Clonal immunoglobulin heavy chain (IGH) rearrangement is usually identified, even when immunoglobulin (Ig) expression is not demonstrable by immunohistochemistry. IGH variable region genes (IGHV) may have somatic hypermutation or may be unmutated with a germline configuration [48]. *c-MYC* plays important roles in cellular proliferation, protein synthesis and apoptosis. About 50% of PBL cases show *MYC* rearrangement, generally with an Ig gene partner, and are associated with MYC protein overexpression by immunohistochemistry [58]. *MYC* amplification and deletion are also detected in 11% and 4% of PBL cases, respectively [49]. *MYC* rearrangement usually occurs in association with a positive EBER status, CD10 expression and *PRDM1* gene mutation and in the absence of mutations in the *MYC* promoter region [37,49,50]. Mutations in the *PRDM1* gene, encoding for the BLIMP1 protein often expressed by PBL, has been found in some cases, and it acts as an MYC regulator [51]. The oncogenic activation of *MYC* in PBL plays an important pathogenetic role in association with EBV infection [49].

### 2.4. Differential Diagnosis

In the current WHO classification, there are distinct lymphomas with plasmablastic morphology and immunophenotype to be considered in the differential diagnosis with PBL, including EC-PEL, EBV-positive DLBCL, NOS, BL, anaplastic lymphoma kinase positive large B-cell lymphoma (ALK-positive LBCL) and plasmablastic myeloma (PM) [8,61,62,63].

PEL, in its solid/extra-cavitary variant, usually involves lymph nodes, although it is reported even at extra-nodal sites, including the GIT. Similar to PBL, EC-PEL occurs often in immunocompromised patients and typically expresses plasma cell-related markers, CD30 and EMA, whereas B-cell antigens are often negative, although more frequently expressed in the extra-cavitary variant. Unlike in PBL usually expressing immunoglobulin (often IgG), cytoplasmic immunoglobulin are generally absent in PEL, although reported with higher expression in EC-PEL. In PEL, EBV infection is present mainly in HIV-positive individuals. KSHV/HHV8 expression in PEL allows for differential diagnosis with PBL, which is consistently HHV8-negative [64].

Unlike PBL, EBV-positive DLBCL, NOS shows a pan B-cell phenotype, strongly expressing CD20 and PAX5, in the absence of plasma cell-associated markers [52,53].

BL shows some similarities with PBL such as frequent occurrence in HIV-positive hosts, the presence of plasmacytic differentiation, EBER positivity and *MYC* rearrangement. However, the phenotype is clearly distinct as BL expresses CD20 and CD10.

ALK-positive LBCL is a high-grade lymphoma usually involving lymph nodes and mediastinum, although extra-nodal sites may be involved. Patients are usually diagnosed in the advanced stage. The neoplastic cells show an immunoblastic or plasmablastic morphology and a rather striking intrasinusoidal growth pattern. Similar to PBL, the neoplastic cells strongly express plasma cell-related markers and EMA and are negative or focally positive for B-cell associated markers and CD45. Unlike PBL, which is often CD30 and EBV positive, ALK-positive DLBCL is usually negative or weakly positive for CD30 and is always EBV-negative. The lymphoma cells are strongly positive for the ALK protein with a restricted granular cytoplasmic staining pattern, which is indicative of t(2;17)(p23;q23) or CTLC-ALK fusion protein. Few cases show cytoplasmic, nuclear and nucleolar ALK staining associated with the NPM-ALK fusion protein [61].

PM and PBL share morphological and immunophenotypic features with the expression of plasma cell-related markers. The presence of IS and a positive EBV status favor PBL. On the other hand, the identification of multiple lytic bone lesions and renal dysfunction plus laboratory data such as monoclonal paraproteinemia, hypercalcemia and light chain proteins in urine support a diagnosis of PM. CD56 and cyclin-D1 expression by neoplastic cells is also in favor of PM. However, in some cases, especially with a solitary lesion, a definitive distinction cannot be made, and according to the current WHO, the use of a descriptive diagnosis such as “plasmablastic neoplasm consistent with PBL or PM” is acceptable [8].

In some PBL cases, the aberrant expression of T-cell markers represents a potential pitfall which that lead to an erroneous diagnosis of T-cell lymphoma.

The main distinguishing features of PBL, PEL and BL are summarized in Table 1.

### 2.5. Treatment and Outcome

PBL shows a dismal outcome with a median survival of 6–11 months. Currently, there is no standard therapy for PBL patients. Due to the frequent CD20 negativity, rituximab has no role in PBL, and the CHOP (cyclophosphamide, doxorubicin, vincristine and prednisone) scheme, used in DLBCL, is considered inadequate. More intensive regimens such as hyper-CVAD-MA (hyper-fractionated cyclophosphamide vincristine, doxorubicin and dexamethasone alternating with methotrexate and cytarabine), CODOX-M/IVAC (cyclophosphamide, vincristine, doxorubicin, methotrexate alternating with ifosfamide, etoposide and cytarabine), COMB (cyclophosphamide, oncovin, methyl-CCNU and bleomycin) and EPOCH (etoposide, prednisone, vincristine, cyclophosphamide and doxorubicin) are used [49]. Further possible therapeutic options include target therapy with the anti-CD30 Brentuximab Vedotin, immunomodulatory drugs such as Bortezomib and Lenalidomide, and autologous hematopoietic stem cell transplantation (auto-HSCT) [49,54,55,56,57]. In HIV-positive patients, a combination of highly active anti-retroviral therapy (HAART) and chemotherapy is used [50]. The majority of HIV-associated PBL harbor *MYC* abnormalities; therefore, targeted therapies of oncogenic *MYC* are considered a potential treatment worthy of evaluation [50]. Both EBV-positive and EBV-negative PBLs show high levels of immune checkpoint protein (PD-1 and PD-L1) expression by the tumor cells and the macrophages of the microenvironment. Therefore, an immune escape process is likely to be present in PBL [60]. Anti-PD-1 agents represent possible new treatment strategies.

## 3. EC-PEL

### 3.1. General Features and Etiology

PEL is an aggressive large B-cell lymphoma classically presenting exclusively as serous effusions in the pleural, pericardial or peritoneal cavities in the absence of an identifiable tumor mass [64]. Although by definition no mass lesion is present in PEL, a contiguous extension of lymphoma into adjacent tissues may occur.

In a minority of cases (approximately 5%), lymphomas indistinguishable for PEL present as solid tumor masses outside body cavities and have been recognized as EC-PEL in the current WHO classification [8,65,66,67,68,69,70,71,72,73,74,75].

KSHV/HHV8 is the pathogen involved in all cases of PEL in both the serous-based classic PEL and EC-PEL; HHV8 detection is a required diagnostic criterion for the disease.

Similar to classic PEL, the extra-cavitary variant most often occurs in HIV-infected individuals or in severe immunodeficiency of some kind. Besides the context of HIV infection, IS may be a consequence of either drugs, for instance following transplantation, or aging [64,76]. The disease may develop in elderly from geographic areas where HHV8 infection is an endemic. Males are affected more frequently than females, and the disease more often arises at younger age in individuals with HIV infection compared with HIV-negative patients (42 vs. 73 years) [8].

In the context of HIV infection, the neoplastic cells are often co-infected by HHV8 and EBV, whereas co-infection is documented more infrequently in HIV-negative cases [8]. EC-PEL occurs as a solitary tumor mass involving preferentially lymph nodes and more rarely extra-nodal sites such as the skin, lungs, CNS and GIT [65,66,67,68,69,70,71,72,73,74,75]. The solid mass may be the only manifestation of EC-PEL [68,72] or may be associated with effusions in the disease course [73,74].

### 3.2. KSHV/HHV8

Similar to EBV, KSHV/HHV8 is a gamma human herpes virus that is lymphotropic and oncogenic.

Unlike EBV, which is ubiquitous, KSHV/HHV8 is endemic in certain geographic areas such as sub-Saharan Africa, Latin America, the Caribbean, the Mediterranean and Middle Eastern countries.

The virus is transmitted through saliva, but it may also be transmitted sexually and even vertically through breast milk. Other possible routes of transmission are blood transfusion, intravenous drug use and transplant [64,76].

Epidemiologic evidence supports a fecal–oral route of spread and viral DNA has been identified in rectal mucosa from HIV-positive individuals [77].

Similar to EBV, HHV8 may infect cells such as endothelial cells and lymphocytes, persisting throughout a person’s life in a latent form. When the host immune control is altered, HHV8 may reactivate the lytic replicative cycle producing viremia [64].

The virus was initially identified as having a pathogenetic role in Kaposi sarcoma (KS) [78], and subsequently, it was identified in HHV8-positive multicentric Castleman’s disease (HHV8-positive MCD) [79], PEL [80] and, recently, in germinotropic lymphoproliferative disorder (GLPD) [81,82,83]. HHV8 is also strictly linked to HHV8-positive diffuse large B-cell lymphoma, not otherwise specified (HHV8-positive DLBCL, NOS), an aggressive lymphoma mainly occurring in MCD patients [8]. With the exception of GLPD, all other KSHV/HHV8-related diseases occur predominantly in immunocompromised individuals.

### 3.3. EC-PEL and GIT

EC-PEL may occur in any part of the GIT, including the anorectal site, although the stomach and large bowel are more often involved [65,66,67,68,69,70,71,72,73,74,75].

The tumor may present as a mass; a polypoid lesion; or a thickening of the gastrointestinal (GI) wall, with or without nodal involvement [65,66,67,68,69,70,71,72,73,74,75].

In GIT, EC-PEL have also been reported before the development of effusions [73] and following the resolution of a classic PEL [74]. Despite the GIT being a reservoir of HHV8, EC-PEL is rarely detected in GIT; this may be explained by the difficulty in correctly identifying this entity unless HHV8 immunostaining is used.

### 3.4. Histology, Immunophenotype and Genetic Profile

The histology of EC-PEL is indistinguishable from classic PEL.

**Figure 4:**
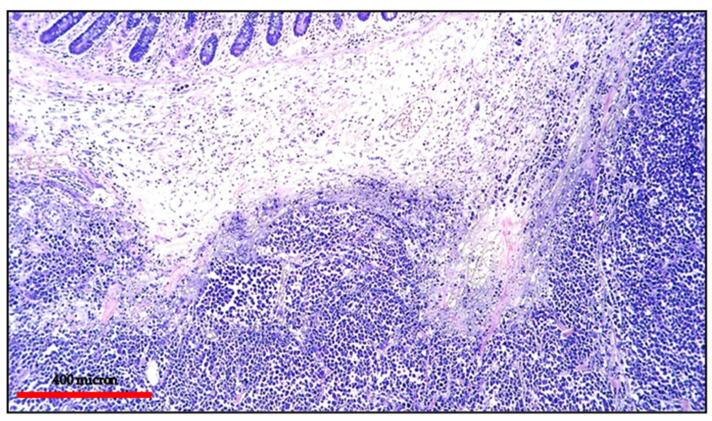
EC-PEL of the ileum: low power view of the neoplastic proliferation involving the bowel wall (Giemsa staining, magnification 100×; original image from Prof Ascani).

**Figure 5:**
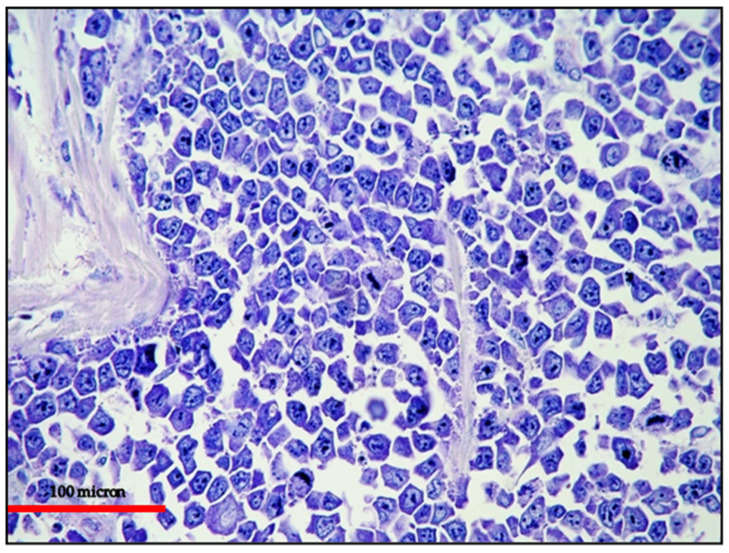
EC-PEL of the ileum: high power highlighting the morphological details of large neoplastic cells with prominent nucleoli (Giemsa staining, magnification 400×; original image from Prof Ascani).

The neoplastic cells are large-sized resembling immunoblasts (IBs) or PBs or displaying an anaplastic morphology (Figure 4 and Figure 5).

PEL in its classic form usually fails to express pan-B-cell markers such as CD19, CD20, CD79α and PAX5 as well as surface and cytoplasmic immunoglobulin. The classic form usually expresses CD45/LCA.

**Figure 6:**
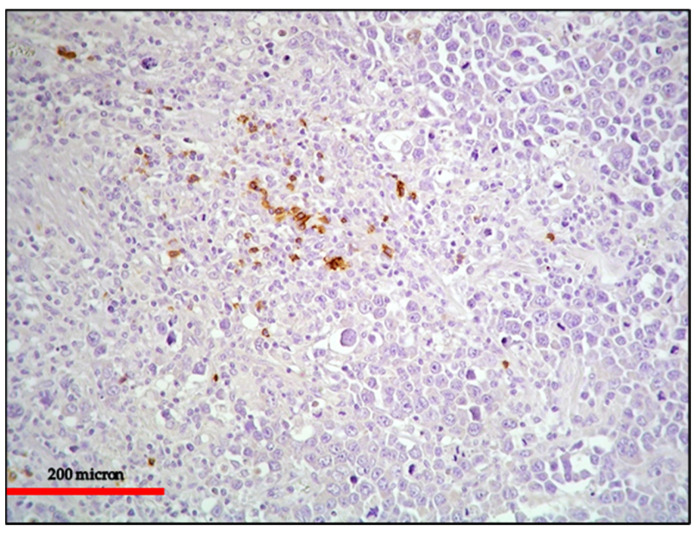
EC-PEL of the ileum: in this case, the neoplastic proliferation lacks B-cell marker expression; this is often a common feature of neoplasms with plasma cell differentiation such as PBL and PEL (CD20 immunostaining, Ventana immunostainer, magnification 200×; original image from Prof Ascani).

The extra-cavitary variant may express B-cell markers (25% vs. <5%), although not in all cases (Figure 6), as well as immunoglobulin (25% vs. 15%) more often than classic PEL and, on the other hand, may have a lower expression of CD45 [70].

**Figure 7:**
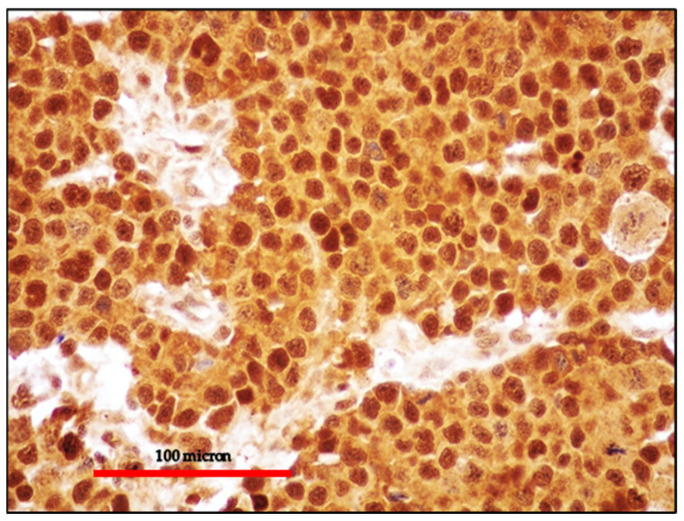
EC-PEL of the ileum: Diffuse and intense expression of MUM18; similar to PBL, EC-PEL expresses markers of plasma cell differentiation (immunostaining, Ventana immunostainer, magnification 400×; original image from Prof Ascani).

The neoplastic cells generally express plasma cell-related markers, including CD138, MUM18 (Figure 7) and MUM1/IRF4, and markers of lymphoid activation, such as CD30, CD38, EMA and HLA-DR. T-cell markers are usually negative. The aberrant expression of T-cell markers may be observed, and it is more frequent in EC-PEL.

**Figure 8:**
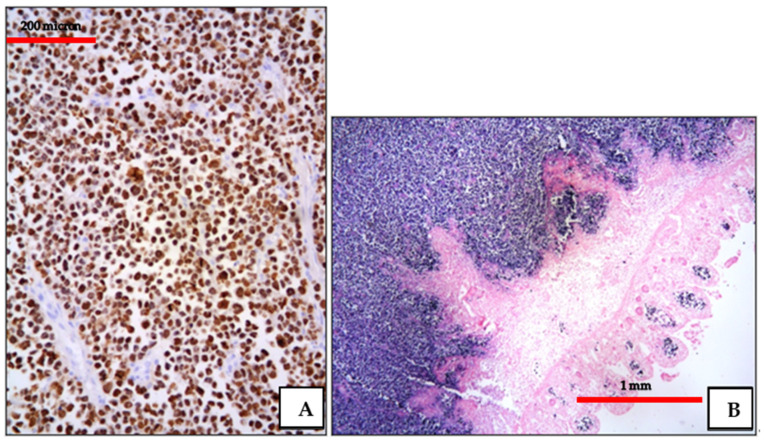
(**A**) EC-PEL of the ileum: strong and diffuse HHV8 positivity of the neoplastic cells; HHV8 expression is a diagnostic feature for EC-PEL (immunostaining, Ventana immunostainer, magnification 200×; original image from Prof Ascani); (**B**) EC-PEL of the ileum: diffuse EBV positivity of the neoplastic proliferation (EBER-ISH, magnification 40×; original image from Prof Ascani).

The lymphoma cells are positive for HHV8 in all cases. HHV8 is usually demonstrated by immunohistochemistry, detecting the expression of HHV8-encoded latency-associated nuclear antigen 1 (LANA-1) protein (Figure 8).

HHV8 can also be documented by in situ hybridization or polymerase chain reaction (PCR) analyses.

EBV co-infection is identified by EBER in 50–80% of PEL. In non-HIV infected individuals, PEL may be EBV-negative. Despite positivity for EBV by EBER, EBV-latent membrane protein 1 (LMP1) is usually absent, showing a type I latency pattern, similar to PBL.

**Figure 9:**
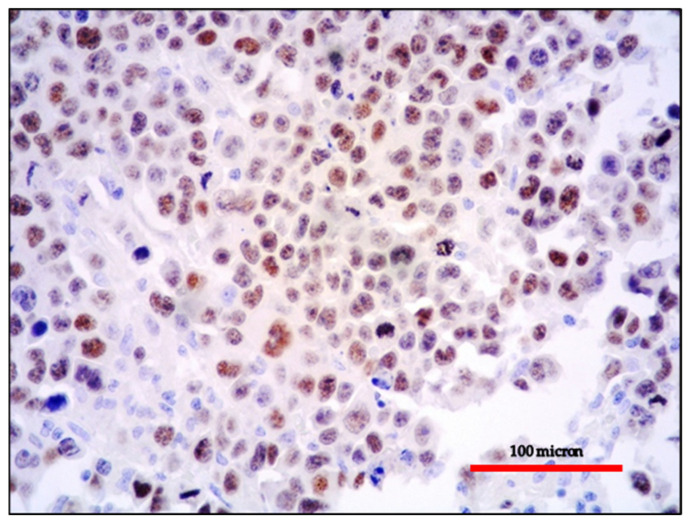
EC-PEL of the ileum: MYC protein expression is frequently found in EC-PEL despite the lack of *MYC* gene alterations (immunostaining, Ventana immunostainer, magnification 400×; original image from Prof Ascani).

Clonal immunoglobulin (IG) gene rearrangement is present, indicating a B-cell origin. In some cases, T-cell receptor (TCR) gene rearrangement may be found in addition to IG genes. HHV8 viral genomes are present in all cases. Rearrangements at the *MYC*, *BCL2* and *BCL6* loci are usually absent. Structural alterations in the *MYC* gene are absent; however, MYC protein overexpression may be seen, probably due to the activity of HHV8-encoded latent proteins [58] (Figure 9).

HHV8-encoded latent proteins, including latency-associated nuclear antigen-1 (LANA-1), LANA-2/vIRF-3, viral cyclin, viral FLICE inhibitory protein (v-FLIP) and kaposin, are considered to play significant roles in PEL. These proteins are related to inhibition of apoptosis (*TP53* and *RB* genes) and activation of the NF-kB, JAK/STAT and PI3K/AKT/mTOR pathways involved in the survival of PEL [58].

### 3.5. Differential Diagnosis

The diagnosis of EC-PEL can be challenging. The difficulty to identify EC-PEL can be explained by the unusual extra-cavitary presentation, the histological features overlapping with other more common lymphomas and the immunophenotype often lacking B-cell markers.

The differential diagnosis is broad and includes anaplastic large cell lymphoma (ALCL); EBV-positive DLBCL, NOS; PBL; HHV8-positive DLBCL, NOS; ALK-positive LBCL; and PM.

The anaplastic morphology and the intrasinusoidal growth observed in some cases of PEL [64] in combination with the possible aberrant T-cell marker expression and strong CD30 positivity may suggest a diagnosis of ALK-negative anaplastic large cell lymphoma (ALCL). Rare cases of intravascular (IV) NK/T cell lymphoma, EBV-positive have been reported. Intravascular lymphoma is more often of B-cell lineage and, rarely, EBV-positive, whereas the rare cases of IV lymphoma of NK/T cell origin are more often EBV-associated. Skin and CNS are more frequently involved, although any site can be affected [84].

Performing a panel of immunostainings including plasma cell-related markers and, in particular, HHV8 helps to avoid missing the diagnosis of EC-PEL; molecular analysis may further support the diagnosis.

Unlike the classic form of PEL, which is usually negative for B-cell markers, EC-PEL often expresses some B-cell markers and should be differentiated from EBV-positive DLBCL, NOS. HHV8 positivity represents the diagnostic criterion for PEL.

The distinction of EC-PEL from PBL has been discussed in the section on PBL.

PEL, in both the classic and extra-cavitary variants, belongs to the spectrum of HHV8-related lymphoid disorders including HHV8-positive MCD [85]; GLPD [81,82,83]; and HHV8-positive DLBCL, NOS [8].

Among these HHV8-linked entities, HHV8-positive DLBCL, NOS is differentially diagnosed with EC-PEL.

HHV8-positive DLBCL, NOS is an aggressive lymphoma usually developing in HIV-positive hosts, mainly in the setting of HHV8-positive MCD and involves lymph nodes, the spleen and the liver, although extra-nodal sites and BM may be affected [8]. It is composed of sheets of PBs, which by definition, are HHV8-positive and EBV-negative, whereas EC-PEL is often, although not always, co-infected by HHV8 and EBV.

The neoplastic cells of HHV8-positive DLBCL, NOS have the same MCD phenotype with characteristic expressions of IgM lambda, MUM1/IRF4 and CD38, and is absent or weak positivity for B-cell markers, CD30 and CD138.

Unlike HHV8-positive DLBCL, NOS, PEL usually expresses CD138, EMA and CD30 and lacks cytoplasmic immunoglobulin.

In contrast with PEL, ALK-positive LBCL is always negative for HHV8 and EBER, and CD30 is negative or only weakly and focally positive; ALK staining confirms the diagnosis of ALK-positive LBCL.

The distinction between PEL and PM frequently relies on clinical correlation. The presence of lytic bone lesions and a serum monoclonal protein favors the diagnosis of PM.

### 3.6. Treatment and Outcome

The outcome of PEL is dismal with a median survival of less than 6 months despite intensive chemotherapy.

EC-PEL in HIV-positive hosts seems to have a slightly better outcome than patients with classic PEL, with a median survival of 11 months vs. 3 months [65]. The study by Lurain et al. reported EBV negativity and high interleukin-6 serum levels as poor prognostic factors [86]. Rituximab is ineffective due to the absence of CD20 expression, and CHOP-based chemotherapy is inadequate; more intensive chemotherapy such as EPOCH, or more recent treatments targeting proteasome, the NF-kB pathway or anti-CD30 therapy are needed, and more studies should be performed [58,87].

## 4. BL

### 4.1. General Features and Etiology

BL is a highly aggressive B-cell lymphoma, named after the surgeon who first identified it in the 1950s in Uganda as a rapidly growing neoplasm in children with jaw and abdominal masses [88,89,90].

In the current WHO classification, there are three epidemiological subtypes of BL: endemic, sporadic and immunodeficiency-related [8].

BL is the first historical example of a virus-associated human neoplasm and shows activation of the *MYC* oncogene [89]. The strength of EBV association with BL varies geographically and, hence, among the different forms of BL.

Endemic BL occurs in certain geographical area (equatorial Africa and Papua New Guinea) with a strict epidemiological correlation with malaria and the EBV genome is identified in >95% of cases of endemic BL [90].

Local environmental factors play a contributory role in BL development [91,92]. In endemic BL, EBV has an etiologic role, acting in synergy with a polymicrobial environment, in particular with *Plasmodium falciparum* and even with other viruses, such as herpesviruses HHV5 and HHV8 [91,92].

Endemic BL is the most common pediatric malignancy in equatorial Africa and Papua New Guinea, with a male predilection (M:F = 2:1) and a median age of 7 years at presentation [89]. The early perinatal EBV infection in these geographical areas probably has a role in the occurrence of endemic BL early in childhood [93].

Sporadic BL is observed worldwide, generally in children and young adults, although three peaks of age at 10, 40 and 70 years are observed; there is a male prevalence (M:F = 2–3:1) [94,95]. In Europe and USA, sporadic BL represents 1–2% of all lymphomas, whereas in certain geographical areas (South America and North Africa), the incidence of sporadic BL is much higher [94,95]. About 20–30% of the sporadic cases are EBV-positive, and EBV-positive sporadic BL are more frequent in adults than in children [94].

Immunodeficiency-related BL occurs in middle-aged HIV-positive patients, often early in the disease course when the count of CD4+ T cells is still high and the use of HAART has not decreased BL incidence [96]. Immunodeficiency-associated BL may also be seen in transplant recipients and individuals with congenital immunodeficiency. EBV is found in about 25−40% of immunodeficiency-associated cases.

BL involves mainly extra-nodal sites with some differences among the three forms.

In endemic BL, the most frequently involved site at presentation is the head and neck area (jaw and facial bones), although the ileocecal region is also frequently involved.

In sporadic BL, the disease more frequently presents as an abdominal mass (especially terminal ileum and cecum) and more uncommonly in the facial region. Other sites involved in both sporadic and endemic forms are the gonads, kidneys and breasts. Lymph node presentation is rare but observed more frequently in adults.

The immunodeficiency-associated BL shows nodal and BM involvement more often. The presentation of the disease as acute leukemia is rare in the endemic form. All of the variants of BL may involve the CNS.

### 4.2. BL and GIT

BL often affects the GIT, although the frequency of GIT involvement varies among the three forms.

Primary GI involvement is observed more frequently in the sporadic form.

The most frequently involved GI site is the small bowel at the ileo-cecal region. The stomach and appendix are more rarely involved.

Due to the significantly elevated proliferation rate of BL, patients generally present with a bulky disease.

BL of the bowel appears as a mass or segmental wall thickening. The intestinal tract involvement usually presents with intussusception, leading to signs of obstruction; bleeding and sometimes malnourishment may be present [97,98]. In the sporadic form, GIT involvement has been found more frequently in EBV-negative cases [94].

### 4.3. Histology, Immunophenotype and Genetic Profile

**Figure 10:**
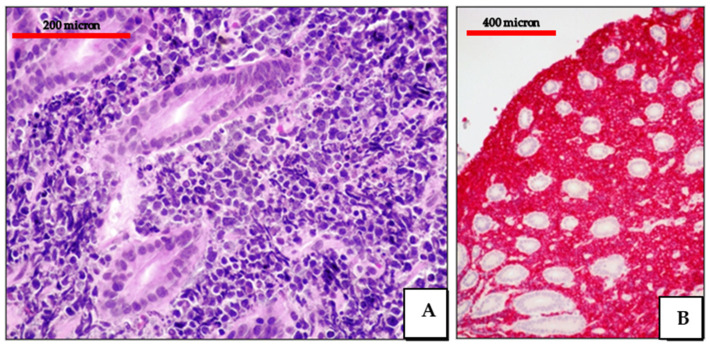
(**A**) BL of the duodenum: lymphoid infiltrate within the duodenal mucosa (Hematoxylin and eosin, magnification 200×; original image from Prof Ascani); (**B**) BL: diffuse CD20 positivity confirming the B-cell phenotype of the lymphoid cells (immunostaining, Ventana immunostainer, magnification 100×; original image from Prof Ascani).

BL typically consists of a diffuse cohesive proliferation of monomorphic medium-sized cells with round nuclei, finely clumped chromatin and multiple small nucleoli. The cytoplasm is deeply basophilic and contains lipid vacuoles more easily seen in imprint preparations (Figure 10).

Frequent mitotic figures and apoptosis are present; the starry sky pattern is indicative of the high proliferative index and is due to the presence of numerous tingible-body macrophages.

Some cases have a more variable morphology with greater nuclear irregularity, whereas a subset of cases show plasmacytic differentiation, especially in HIV-positive patients.

**Figure 11:**
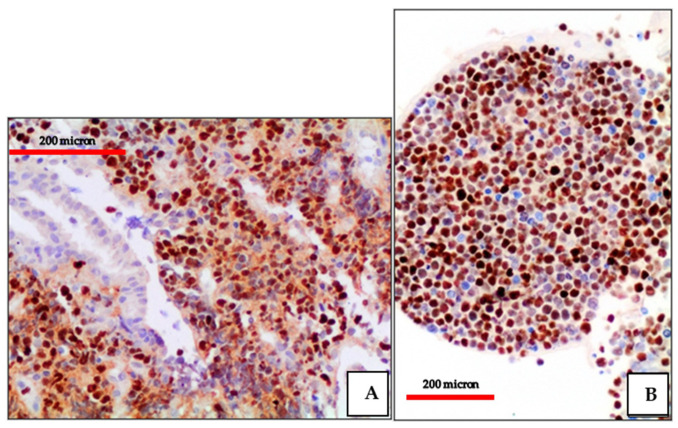
(**A**) BL of the duodenum: diffuse EBV expression by in situ hybridization (EBER-ISH, magnification 200×; original image from Prof Ascani); (**B**) BL of the duodenum: diffuse MYC protein overexpression of the lymphoid cells (immunostaining, Ventana immunostainer, magnification 200× original image from Prof Ascani).

BL cells have a full B-cell phenotype with strong positivity for CD20, CD79alpha, PAX5, CD19 and IgM expression with light chain restriction. The cells express germinal center markers (CD10 and BCL6) and are usually negative for BCL2. In some cases, there is an atypical phenotype with the absence of CD10, CD5 positivity and weak BCL2 expression. The proliferation rate is very high (ki67 > 95%) and there is strong MYC protein overexpression. EBER is positive (Figure 11) in the majority of endemic BL and only in a subset of sporadic and immunodeficiency-associated cases [8]. 

The reciprocal chromosomal translocation between the *MYC* proto-oncogene and one of the IG genes is identified in BL, irrespective of the epidemiological form and of whether or not BL carries EBV.

However, *MYC* translocations may occur even in other types of lymphoma and are not specific of BL.

A breakpoint in the long arm of chromosome 8 at the 8q24 locus, next to or within the *MYC* gene, is involved in the three most common translocations. In the majority of BL (about 80%), there is the translocation (t(8;14)(q24;q32)) that transposes the telomeric region of chromosome 8 to the IGH on chromosome 14.

Other translocations such as t(2;8) (p12;q24) and t(8;22) (q24;q11) are present in a subset of cases.

In sporadic and immunodeficiency-related BL, the breakpoints are usually within or close to *MYC*, whereas in the endemic form, the breakpoints are dispersed several hundred kilobases further upstream of the gene [99,100]. About 10% of BL cases lack an identifiable *MYC* rearrangement [8]. According to the current WHO classification in these *MYC*-negative cases, stringent clinical, morphological and immunophenotypic criteria need to be used to rule out lymphomas that may simulate BL. Some of these cases lacking *MYC* rearrangement represent the provisional entity Burkitt-like lymphoma with 11 q aberration [8].

From the activation of oncogenes such as *MYC*, some direct consequences are cell growth and proliferation, which may cause lymphoma genesis [99].

However, activated *MYC* may also induce cell death by apoptosis and an irreversible block to proliferation named senescence [99]. Lymphoma may develop only if these latter processes are repressed.

EBV promotes epigenetic changes of the proapoptotic protein named Bim, inhibiting Bim function and, hence, favoring cell survival [99,100]. In BL, EBV shows the latency pattern I. This represents the most restricted latency pattern with only the expression of EBNA-1. EBV shows a synergic function with B lymphoid cells harboring a *MYC* translocation, leading to the inhibition of the proapoptotic signaling [99,100].

### 4.4. Differential Diagnosis

BL need to be differentiated from high-grade B-cell lymphoma with *MYC* and *BCL2* and/or *BCL6* rearrangements, so called double-hit and triple-hit lymphomas.

A subset of these lymphomas (about 50%) shows morphological features closely mimicking BL or has features in between DLBCL and BL.

The diffuse proliferation of monomorphic, medium-sized cells simulates BL although the cytoplasm is probably less basophilic than those in BL and the cytoplasmic vacuoles are absent. BL can be ruled on the basis of the immunophenotype (often strong BCL2 expression in contrast to the absent or weak BCL2 expression in BL) and molecular genetic findings.

A subset of BL shows plasmacytic features and needs to be distinguished from other lymphomas with plasma cell differentiation such as PBL and PEL. Unlike these neoplasms often lacking B-cell markers, BL shows a pan-B-cell phenotype.

Of note, the provisional entity of Burkitt-like lymphoma with 11 q aberration has to be excluded in cases resembling BL but lacking *MYC* rearrangements. Compared with BL, this subset of lymphomas often involves lymph nodes and shows a certain degree of nuclear pleomorphism.

### 4.5. Treatment and Outcome

Current treatments employ intensive, multi-agent regimens that have significantly improved the overall survival of BL patients to almost 90% of cases [101].

The chemotherapeutic schemes involve CODOX-M/IVAC (cyclophosphamide, vincristine, doxorubicin, high-dose methotrexate/ifosfamide, etoposide and high-dose cytarabine) and DA-EPOCH-R (etoposide, prednisone, vincristine, cyclophosphamide, doxorubicin and rituximab). Modified Berlin–Frankfurt–Münster (BMF) chemotherapeutic regimens plus rituximab have been adopted in adult patients affected by BL, with high rates of complete response (CR) and durable responses [102]. Due to the high risk of CNS involvement, CNS prophylaxis with intrathecal methotrexate and/or cytarabine is always performed. The tumor lysis syndrome (TLS) is a potential complication of high-dose intensive chemotherapy due to rapid tumor growth. CNS and BM involvement are associated with worse prognosis.

## 5. Conclusions

Clinicians and pathologists should be aware of the complex and expanding spectrum of EBV-linked LPDs that may arise even in the GIT. These entities are often difficult to correctly diagnose not only due to their rarity but also due to their overlapping morphologic and immunophenotypic features shared by different entities.

Their diagnoses should be based on the evaluation of multiple parameters such as clinical data, morphology, immunophenotyping and genetic analysis.

## Figures and Tables

**Table 1:** Clinicopathologic features of PEL, PBL and BL.

	PEL	PBL	BL
**Immunosuppression**	Usually present	Usually present	Present in the immunodeficiency-associated variant
**HIV status**	+ (− in elderly and EBV-negative cases)	+ (often); in HIV negative pts other causes of IS are present.	+ in the immunodeficiency-associated variant
**Clinical presentation**	Effusion in its classic form. Extra-nodal sites (mostly) and nodal sites in EC-PEL	Extra-nodal sites frequent; rarely nodal sites	Extra-nodal sites mostly; nodal sites in the Immunodeficiency associated variant
**Histology**	PBs/IBs in fluids in the classic form; sheets of PBs/IBs in EC-PEL	PBs/IBs (diffuse pattern of growth)	Medium-sized cells (diffuse monotonous pattern of growth)
**CD45**	+ (often − in EC-PEL)	− or weakly + in a minority of cells	+
**CD20**	− (often + in EC-PEL)	− or weakly + in a minority of cells	+
**PAX5**	− (often + in EC-PEL)	− or weakly + in a minority of cells	+
**CD79α**	− (often + in EC-PEL)	+ in about 40% of cases	+
**MUM1/IRF4**	+	+	−/+
**CD10**	-	− (+ in 20%)	+
**BCL6**	-	-	+
**BCL2**	+ often	-	− (rarely weakly +)
**CD38**	+	+	+ often
**CD138**	+	+	-
**CD30**	+	+	-
**EMA**	Often +	+	-
**MYC protein**	May be + (due to HHV8-encoded latent proteins)	+ (more often in EBV+ cases)	+
**T cell markers**	Occasionally + (more often in EC-PEL)	Occasionally +	-
**Light chain restriction**	Absent (often + in EC-PEL)	+ (often IgG kappa or lambda)	+ (IgM kappa or lambda)
**HHV8**	+	-	-
**EBV** **(by EBER-ISH)**	+ (− in elderly HIV-negative pts)	+ in HIV-positive pts and post-transplant pts	>95% endemic variant; 20–30% sporadic variant; 25–40% immunodeficiency-associated variant
***MYC*** **rearrangements**	absent	Present in 50% of cases (mainly in EBV-positive cases)	+ in 90% of cases
**Clonality (Ig genes)**	Monoclonal (IgG genes hypermutated)	Monoclonal	Monoclonal
**Outcome**	Poor	Poor	Highly aggressive, but potentially curable

EBV: Epstein–Barr-virus; EC-PEL: extra-cavitary PEL; EBER-ISH: EBV-encoded RNA in situ hybridization; HIV: human immunodeficiency virus; IBs: immunoblasts; Ig: immunoglobulin; PBs: plasmablasts; PBL: plasmablastic lymphoma; PEL: primary effusion lymphoma; pts: patients.

## Data Availability

Individual patient data from the original studies included in this review are not available, and data sharing at this level is not applicable for a systematic review.

## Supplementary Materials

The following are available online at https://www.mdpi.com/article/10.3390/cancers13184527/s1, Table S1: EBV-positive Lymphoproliferative disorders.

## References

[1] Taylor G.S., Long H.M., Brooks J.M., Rickinson A.B., Hislop A.D. (2015). The immunology of Epstein-Barr virus induced disease. Annu. Rev. Immunol..

[2] Young L.S., Yap L.F., Murray P.G. (2016). Epstein-Barr virus: More than 50 years old and still providing surprises. Nat. Rev. Cancer.

[3] Resk S.A., Weiss L.M. (2007). Epstein-Barr virus-associated lymphoproliferative disorders. Hum. Pathol..

[4] Tanner J.E., Alfieri C. (2001). The Epstein-Barr virus and post-transplant lymphoproliferative disease: Interplay of immunosuppression, EBV, and the immune system in disease pathogenesis. Transpl. Infect. Dis..

[5] Hakim F.T., Gress R.E. (2007). Immunosenescence: Deficits in adaptative immunity in the elderly. Tissue Antigens.

[6] Ghia P., Prato G., Stella S., Scielzo C., Geuna M., Caligaris-Cappio F. (2007). Age-dependent accumulation of monoclonal CD4+ CD8+ double positive T lymphocytes in the peripheral blood of the elderly. Br. J. Haematol..

[7] Dolcetti R., Dal Col J., Martorelli D., Carbone A., Klein E. (2013). Interplay among viral antigens, cellular pathways and tumor microenvironment in the pathogenesis of EBV-driven lymphoma. Semin. Cancer Biol..

[8] Swerdlow S.H., Campo E., Harris N.L., Jaffe E.S., Pileri S.A., Stein H., Thiele J. (2017). WHO Classification of Tumours Haematopoietic and Lymphoid Tissues.

[9] Dojcinov S.D., Fend F., Quintanilla-Martinez L. (2021). EBV-positive lymphoproliferation of B-T- and NK-cell derivation in non-immunocompromised hosts. Pathogens.

[10] Dunmire S.K., Hogquist K.A., Balfour H.H. (2015). Infectious mononucleosis. Curr. Top. Microbiol..

[11] Rickinson A.B. (2014). Co-infections, inflammation and oncogenesis: Future directions for EBV research. Seminars in Cancer Biology.

[12] Price A.M., Luftig M.A. (2015). To be or not IIb: A multi-step process for Epstein-Barr virus latency establishment and consequences for B cell tumorigenesis. PLoS Pathog..

[13] Dojcinov S.D., Venkataraman G., Raffeld M., Pittaluga S., Jaffe E.S. (2010). EBV positive mucocutaneous ulcer. A study of 26 cases associated with various sources of immunosuppression. Am. J. Surg. Pathol..

[14] Ikeda T., Gion Y., Yoshino T., Sato Y. (2019). A review of EBV-positive mucocutaneous ulcers focusing on clinical and pathological aspects. J. Clin. Exp. Hematopath..

[15] Ikeda T., Gion Y., Nishimura M.F., Yoshino T., Sato Y. (2021). Epstein-Barr Virus-positive mucocutaneous ulcer: A unique and curious disease entity. Int. J. Mol. Sci..

[16] Natkunam Y., Goodlad J.R., Chadburn A., Jong D., Gratzinger D., Chan J.K.C., Said J., Jaffe E.S. (2017). EBV-positive B-cell proliferations of varied malignant potential. Am. J. Clin. Pathol..

[17] Hart M., Thakral B., Yohe S., Balfour H.H., Singh C., Speras M., McKenna R.W. (2014). EBV-positive mucocutaneous ulcer in organ transplant recipients: A localized indolent posttransplant lymphoproliferative disorder. Am. J. Surg. Pathol..

[18] Matnani R., Peker D. (2014). Azathioprine induced Epstein Barr virus-positive mucocutaneous ulcer arising in perianal fistula and abscess associated with Crohn’s disease. J. Crohn’s Colitis.

[19] Moran N.R., Webster B., Lee K.M., Trotman J., Kwan Y.-L., Napoli J., Leong R.W. (2015). Epstein Barr virus-positive mucocutaneous ulcer of the colon associated Hodgkin lymphoma in Crohn’s disease. World J. Gastroenterol..

[20] Juan A., Lobaton T., Tapja G., Manosa M., Cabrè E. (2017). Epstein-Barr virus-positive mucocutaneous ulcer in Crohn’s disease. A condition to consider in immunosuppressed IBD patients. Dig. Liver Dis..

[21] Zanelli M., Mengoli M.C., Valli R., Froio E., Bisagni A., Zizzo M., De Marco L., Ascani S. (2019). Primary classic Hodgkin lymphoma of the ileum and Epstein-Barr virus mucocutaneous ulcer of the colon: Two entities compared. Virchows Arch..

[22] Kleinman S., Jhaveri D., Caimi P., Cameron R., Lemonovich T., Meyerson H., Hostoffer R., Tcheurekdjian H. (2014). A rare presentation of EBV+ mucocutaneous ulcer that led to the diagnosis of hypogammaglobulinemia. J. Allergy Clin. Immunol. Pract..

[23] Osman M., Al Salihi M., Abu Sitta E., Al Hadidi S. (2017). A rare case of Epstein-Barr virus mucocutaneous ulcer of the colon. BMJ Case Rep..

[24] Zanelli M., Zizzo M., Foroni M., De Marco L., Martino G., Ascani S. (2019). EBV-positive mucocutaneous ulcer within colonic diverticulitis mimicking diffuse large B-cell lymphoma. Ann. Hematol..

[25] Volaric A.K., Singh K., Gru A.A. (2021). Rare EBV-associated B cell neoplasms of the gastrointestinal tract. Semin. Diagn. Pathol..

[26] Ikeda T., Gion Y., Sakamoto M., Tachibana T., Nishikori A., Nishimura M.F., Yoshino T., Sato Y. (2020). Clinicopathological analysis of 34 Japanese patients with EBV-positive mucocutaneous ulcer. Mod. Pathol..

[27] Kumar S., Fend F., Quintanilla-Martinez L., Kingma D., Sorbara L., Raffeld M., Banks P.M., Jaffe E.S. (2000). Epstein-Barr virus-positive primary gastrointestinal Hodgkin’s disease: Association with inflammatory bowel disease and immunosuppression. Am. J. Surg. Pathol..

[28] Barzilai M., Polliack A., Avivi I., Herishanu Y., Ram R., Tang C., Perry C., Sarid N. (2018). Hodgkin lymphoma of the gastrointestinal tract in patients with inflammatory bowel disease: Portrait of a rare entity. Leuk. Res..

[29] Gibson B., Podoll M.B., Baumgartner E.M., Maley D.H. (2016). Syncitial variant of nodular sclerosis classical Hodgkin lymphoma of the terminal ileum in a patient with longstanding Crohn’s disease. Ann. Clin. Lab. Sci..

[30] Rezk S.A., Weiss L.M. (2019). EBV-associated lymphoproliferative disorders: Update in classification. Surg. Pathol..

[31] Chen A., Donati D., Orem J., Mbidde E.R., Kironde F., Wahlgren M., Bejarano M.T. (2009). Endemic Burkitt’s lymphoma as a polymicrobial disease: New insights on the interaction between Plasmodium falciparum and Epstein-Barr virus. Semin. Cancer Biol..

[32] Mack A.A., Sugden B. (2008). EBV is necessary for proliferation of dually infected primary effusion lymphoma cells. Cancer Res..

[33] Swerdlow S.H., Campo E., Harris N.L., Jaffe E.S., Pileri S.A., Stein H., Thiele J. (2008). WHO Classification of Tumours of Haematopoietic and Lymphoid Tissue.

[34] Delecleuse H.J., Anagnostopoulos I., Dallenbach F., Hummel M., Marafioti T., Schneider U., Huhn D., Schmidt-Westhausen A., Reichart P.A., Gross U. (1997). Plasmablastic lymphomas of the oral cavity: A new entity associated with the human immunodeficiency virus infection. Blood.

[35] Zizzo M., Zanelli M., Martiniani R., Sanguedolce F., De Marco L., Martino G., Parente P., Annessi V., Manzini L., Ascani S. (2020). Oral plasmablastic lymphoma: A case report. Medicine.

[36] Morscio J., Dierickx D., Nijs J., Verhoef G., Bittoun E., Vanoeteren X., Wlodarska I., Sagaert X., Tousseyn T. (2014). Clinicopathologic comparison of plasmablastic lymphoma in HIV-positive, immunocompetent and posttransplant patients single-center series of 25 cases and meta-analysis of 277 reported cases. Am. J. Surg. Pathol..

[37] Castillo J.J., Bibas M., Miranda R.N. (2015). The biology and treatment of plasmablastic lymphoma. Blood.

[38] Harmon C.M., Smith L.B. (2016). Plasmablastic lymphoma: A review of clinicopathologic features and differential diagnosis. Arch. Pathol. Lab. Med..

[39] Sanguedolce F., Zanelli M., Zizzo M., Martino G., Rossi C., Parente P., Ascani S. (2020). Clinical, pathological and molecular features of plasmablastic lymphoma arising in the gastrointestinal tract: A review and reappraisal. Pathol. Res. Pract..

[40] Hansen K.D., Sabuncyan S., Langmead B., Nagy N., Curley R., Klein G., Klein E., Salamon D., Feinberg A.P. (2014). Large-scale hypomethylated blocks associated with Epstein-Barr virus-induced B-cell immortalization. Genome Res..

[41] Lopez A., Abrisqueta P. (2018). Plasmablastic lymphoma: Current perspectives. Blood Lymph. Cancer Targets Ther..

[42] Foukas P.G., de Leval L. (2015). Recent advances in intestinal lymphomas. Histopathology.

[43] Luria L., Nguyen J., Zhou J., Jaglal M., Sokol L., Messina J.L., Coppola D., Zhang L. (2014). Manifestations of gastrointestinal plasmablastic lymphoma: A case series with literature review. World J. Gastroenterol..

[44] Bewtra M., Lewis J.D. (2010). Update on the risk of lymphoma following immunosuppressive therapy for inflammatory bowel disease. Expert Rev. Clin. Immunol..

[45] Subramaniam K., D’Rozario J., Pavli P. (2013). Lymphoma and other lymphoproliferative disorders in inflammatory bowel disease: A review. J. Gastroenterol. Hepatol..

[46] Liu C., Varikatt W., Ping C.H. (2014). Plasmablastic lymphoma presenting as a colonic stricture in Crohn’s disease. Pathology.

[47] Zanelli M., Ragazzi M., Valli R., De Marco L., Cecinato P., Azzolini F., Ferrari A., Bacci F., Ascani S. (2015). Unique presentation of a plasmablastic lymphoma superficially involving the entire large bowel. Pathol. Res. Pract..

[48] Gaidano G., Cerri M., Capello D., Berra E., Deambrogi C., Rossi D., Larocca L.M., Campo E., Gloghini A., Tirelli U. (2002). Molecular histogenesis of plasmablastic lymphoma of the oral cavity. Br. J. Haematol..

[49] Valera A., Balague O., Colomo L., Martinez A., Delabie J., Taddesse-Heath L., Jaffe E.S., Campo E. (2010). IG/*MYC* rearrangements are the main cytogenetic alteration in plasmablastic lymphomas. Am. J. Surg. Pathol..

[50] Pather S., Mashele T., Willem P., Patel M., Perner Y., Motaung M., Nagiah N., Waja F., Philip V., Lakha A. (2021). *MYC* status in HIV-associated plasmablastic lymphoma: Dual colour CISH, FISH and immunohistochemistry. Histopathology.

[51] Montes-Moreno S., Martinez-Magunacelaya N., Zecchini-Barrese T., Gonzalez de Villambrosia S., Linares E., Ranchal T., Rodriguez-Pinilla M., Battle A., Cereceda-Company L., Revert-Arce J.B. (2017). Plasmablastic lymphoma phenotype is determined by genetic alterations in *MYC* and *PRDM1*. Mod. Pathol..

[52] Castillo J.J., Beltran B.E., Miranda R.N., Young K.H., Chavez J.C., Sotomayor E.M. (2018). EBV-positive large B-cell lymphoma, not otherwise specified: 2018 update on diagnosis, risk-stratification and management. Am. J. Hematol..

[53] Ishikawa E., Satou A., Nakamura M., Nakamura S., Fujishiro M. (2021). Epstein-Barr virus positive B-cell lymphoproliferative disorder of the gastrointestinal tract. Cancers.

[54] Castillo J.J. (2011). Plasmablastic lymphoma: Are more intensive regimens needed?. Leuk. Res..

[55] Pretscher D., Kalish A., Wilhelm M., Birkmann J. (2017). Refractory plasmablastic lymphoma. A review of treatment options beyond standard therapy. Ann. Hematol..

[56] Castillo J.J., Reagan J.L., Sikov W.M., Winer E.S. (2015). Bortezomib in combination with infusional dose-adjusted EPOCH for the treatment of plasmablastic lymphoma. Br. J. Haematol..

[57] Al-Malki M.M., Castillo J.J., Sloan J.M., Re A. (2014). Hematopoietic cell transplantation for plasmablastic lymphoma: A review. Biol. Blood Marrow Transplant..

[58] Chen B.J., Chuang S.S. (2020). Lymphoid neoplasms with plasmablastic differentiation: A comprehensive review and diagnostic approaches. Adv. Anat. Pathol..

[59] Loghavi S., Alayed K., Aladily T.N., Zuo Z., Ng S.B., Tang G., Hu S., Yin C.C., Miranda R.N., Medeiros L.J. (2015). Stage, age and EBV status impact outcomes of plasmablastic lymphoma patients: A clinicopathologic analysis of 61 patients. J. Hematol. Oncol..

[60] Laurent C., Fabiani B., Do C., Tchernonog E., Cartron G., Gravelle P., Amara N., Malot S., Palisoc M.M., Copie-Bergman C. (2016). Immune-checkpoint expression in Epstein-Barr virus positive and negative plasmablastic lymphoma: A clinical and pathological study in 82 patients. Haematologica.

[61] Zanelli M., Valli R., Capodanno I., Ragazzi M., Ascani S. (2015). Anaplastic lymphoma kinase-positive large B-cell lymphoma: Description of a case with an unexpected clinical outcome. Int. J. Surg. Pathol..

[62] Zanelli M., Mengoli M.C., Fanni D., Froio E., De Marco L., Ascani S. (2018). Multiple myeloma with multilobated plasma cells: An unusual and challenging morphological variant. Int. J. Surg. Pathol..

[63] Zanelli M., Ricci S., Zizzo M., Sanguedolce F., De Giorgi F., Palicelli A., Martino G., Ascani S. (2021). Systemic mastocytosis associated with “smoldering” multiple myeloma. Diagnostics.

[64] Zanelli M., Sanguedolce F., Zizzo M., Palicelli A., Bassi M.C., Santandrea G., Martino G., Soriano A., Caprera C., Corsi M. (2021). Primary effusion lymphoma occurring in the setting of transplanted patients: A systematic review of a rare, life-threatening post-transplantation occurrence. BMC Cancer.

[65] Chadburn A., Hyjek E., Mathew S., Cesarman E., Said J., Knowles D.M. (2004). KSHV-positive solid lymphomas represent an extra-cavitary variant of primary effusion lymphoma. Am. J. Surg. Pathol..

[66] Kim Y., Leventaki V., Bhaijee F., Jackson C.C., Medeiros L.J., Vega F. (2012). Extracavitary/solid variant of primary effusion lymphoma. Ann. Diagn. Pathol..

[67] Pan Z.G., Zhang Q.Y., Lu Z.B.L., Quinto T., Rozenvald I.B., Liu L.T., Wilson D., Reddy V., Huang Q., Wang H.Y. (2012). Extracavitary KSHV-associated large B-cell lymphoma: A distinct entity or a subtype of primary effusion lymphoma? Study of 9 cases and review of an additional 43 cases. Am. J. Surg. Pathol..

[68] Costes V., Faumont N., Cesarman E., Rousset T., Meggetto F., Delsol G., Brousset P. (2002). Human herpes-virus-8-associated lymphoma of the bowel in human immunodeficiency virus-positive patients without history of primary effusion lymphoma. Hum. Pathol..

[69] Navarro J.T., Ribera J.M., Juncà J., Millà F. (2003). Anorectal lymphoma without effusion associated with human herpesvirus-8 and type 1 Epstein-Barr virus in an HIV infected patient. Hum. Pathol..

[70] Liao G., Cai J., Yue C., Quing X. (2015). Extracavitary/solid variant of primary effusion lymphoma presenting as a gastric mass. Exp. Mol. Pathol..

[71] Pantanowitz L., Wu Z., Dezube B.J., Pihan G. (2005). Extracavitary primary effusion lymphoma of the anorectum. Clin. Lymphoma Myeloma.

[72] Zanelli M., Bertuzzi C., Zizzo M., Martino G., Sabattini E., Ascani S. (2019). Extracavitary primary effusion lymphoma in a post-transplantation patient. Br. J. Hematol..

[73] DePond W., Said J.W., Tasaka T., de Vos S., Kahn D., Cesarman E., Knowles D.M., Koeffler H.P. (1997). Kaposi’s sarcoma-associated herpesvirus and human herpesvirus 8 (KSHV/HHV8)-associated lymphoma of the bowel. Report of two cases in HIV-positive men with secondary effusion lymphomas. Am. J. Surg. Pathol..

[74] Huang Q., Chang K.L., Gaal K., Aber D.A. (2002). Primary effusion lymphoma with subsequent development of a small bowel mass in an HIV-seropositive patient: A case report and literature review. Am. J. Surg. Pathol..

[75] Oster C., Stein T., Kitahara S., Alkan S., Huang Q. (2018). Kaposi sarcoma-associated herpesvirus/human herpesvirus 8-associated extracavitary primary effusion lymphoma presenting as multiple lymphomatous polyposis. Hum. Pathol..

[76] Pellet Madan R., Hand J. (2019). Human herpesvirus 6, 7, and 8 in solid organ transplantation: Guidelines from the American Society of Transplantation Infectious Diseases Community of Practice. Clin. Transplant..

[77] Thomas J.A., Brookes L.A., McGown I., Weller I., Crawford D.H. (1996). HHV8 DNA in normal gastrointestinal mucosa from HIV seropositive people. Lancet.

[78] Chang Y., Cesarman E., Pessin M., Lee F., Culpepper J., Knowels D.M., Moore P.S. (1994). Identification of herpesvirus-like DNA sequences in AIDS-associated Kaposi’s sarcoma. Science.

[79] Soulier J., Grollet L., Oksenhendler E., Cacoub P., Cazals-Hatem D., Babinet P., d’Agay M.F., Clauvel J.P., Raphael M., Degos L. (1995). Kaposi’s sarcoma-associated herpesvirus-like DNA sequences in multicentric Castleman’s disease. Blood.

[80] Cesarman E., Chang Y., Moore P.S., Said J.W., Knowles D.M. (1995). Kaposi’s sarcoma-associated herpesvirus-like DNA sequences in AIDS-related body cavity-based lymphomas. N. Engl. J. Med..

[81] Du M.Q., Diss T.C., Liu H., Ye H., Hamoudi R.A., Cabecadas J., Dong H.Y., Lee Harris N., Chan J.K.C., Rees J.W. (2002). KSHV and EBV-associated germinotropic lymphoproliferative disorder. Blood.

[82] Zanelli M., Zizzo M., Bisagni A., Froio E., De Marco L., Valli R., Filosa A., Luminari S., Martino G., Massaro F. (2020). Germinotropic lymphoproliferative disorder: A systematic review. Ann. Hematol..

[83] Zanelli M., Fraternali Orcioni G., Zizzo M., De Marco L., Martino G., Cerrone G., Cabra A.D., Ascani S. (2019). HHV-8- and EBV-positive germinotropic lymphoproliferative disorder. Ann. Hematol..

[84] Zanelli M., Mengoli M.C., Del Sordo R., Cagini A., DE Marco L., Simonetti E., Martino G., Zizzo M., Ascani S. (2018). Intravascular NK/T-cell lymphoma, Epstein-Barr virus positive with multiorgan involvement; a clinical dilemma. BMC Cancer.

[85] Zanelli M., Stingeni L., Zizzo M., Martino G., Sanguedolce F., Marra A., Crescenzi B., Pileri S.A. (2021). HHV8-positive Castleman disease and in situ mantle cell neoplasia within dermatopathic lymphadenitis, in longstanding psoriasis. Diagnostics.

[86] Lurain K., Polizzotto M.N., Aleman K., Bhutani M., Wyvill K.M., Goncalves P.H., Ramaswami R., Marshall V.A., Miley W., Steinberg S.M. (2019). Viral, immunologic and clinical features of primary effusion lymphoma. Blood.

[87] Okada S., Goto H., Yotsumoto M. (2014). Current status of treatment for primary effusion lymphoma. Intractable Rare Dis. Res..

[88] Burkitt D. (1958). A sarcoma involving the jaw of African children. Br. J. Surg..

[89] Epstein M.A., Achong B.G., Barr Y.M. (1964). Virus particle in cultured lymphoblasts from Burkitt’s lymphoma. Lancet.

[90] De Thé G., Geser A., Day N.E., Tukei P.M., Williams E.H., Beri D.P., Smith P.G., Dean A.G., Bronkamm G.W., Feorino P. (1978). Epidemiological evidence for causal relationship between Epstein-Barr virus and Burkitt’s lymphoma from Ugandan prospective study. Nature.

[91] Van Den Bosch C. (2012). A role for RNA viruses in the pathogenesis of Burkitt’s lymphoma: The need for reappraisal. Adv. Hematol..

[92] Abate F., Ambrosio M.R., Mundo L., Laginestra M.A., Fulingni F., Rossi M., Zairis S., Gazaneo S., De Falco G., Lazzi S. (2015). Distinct viral and mutational spectrum of endemic Burkitt lymphoma. PLoS Pathog..

[93] De Thè G. (1977). Is Burkitt’s lymphoma related to perinatal infection by Epstein-Barr virus?. Lancet.

[94] Satou A., Asano N., Nakazawa A., Osumi T., Tsurusawa M., Ishiguro A., Elsayed A.A., Nakamura N., Ohshima K., Kinoshita T. (2015). Epstein-Barr virus (EBV)-positive sporadic Burkitt lymphoma: And age-related lymphoproliferative disorder?. Am. J. Surg. Pathol..

[95] Mbulaiteye S.M., Anderson W.F., Ferlay J., Bhatia K., Chang C., Rosenberg P.S., Devesa S.S., Parkin D.M. (2012). Pediatric, elderly and emerging adult-onset peaks in Burkitt’s lymphoma incidence diagnosed in four continents, excluding Africa. Am. J. Hematol..

[96] Gibson T.M., Morton L.M., Shiels M.S., Clarke C.A., Engels E.A. (2014). Risk of non-Hodgkin lymphoma subtypes in HIV-infected people during the HAART era: A population-based study. AIDS.

[97] Molyneux E.M., Rochford R., Griffin B., Newton R., Jackson G., Menon G., Harrison C.J., Israels T., Bailey S. (2012). Burkitt’s lymphoma. Lancet.

[98] England R.J., Oillay K., Davidson A., Numanoglu A., Millar A.J. (2012). Intussusception as a presenting feature of Burkitt lymphoma: Implications for management and outcome. Pediatr. Surg. Int..

[99] Rowe M., Kelly G.L., Bell A.I., Rickinson A.B. (2009). Burkitt’s lymphoma: The Rosetta stone deciphering Epstein-Barr virus biology. Semin. Cancer Biol..

[100] Allday M.J. (2009). How does Epstein-Barr virus (EBV) complement the activation of MYC in the pathogenesis of Burkitt’s lymphoma?. Semin. Cancer Biol..

[101] Duleavy K., Little R.F., Wilson W.H. (2016). Update on Burkitt lymphoma. Hematol. Oncol. Clin. North Am..

[102] Harris E., Paneesha S., Jackson N., Jones L., Mahendra P. (2002). Burkitt’s lymphoma: Single centre experience with modified BMF protocol. Clin. Lab. Haematol..

