# Supplementary material for: EBV-Driven Lymphoproliferative Disorders and Lymphomas of the Gastrointestinal Tract: A Spectrum of Entities with a Common Denominator (Part 2)
Source: Cancers (Basel). 2021 Sep 8;13(18):4527. doi: 10.3390/cancers13184527 (PMC8469260; doi:10.3390/cancers13184527)
Supplement: Supplementary file 1 [file cancers-13-04527-s001.zip › cancers-1369703-supplementary.pdf]

Table S1. EBV-positive Lymphoproliferative disorders.

	IS	HIV status	Sites involved	GIT	Histology	Cell of origin	EBV by EBER	HHV8	Genetic profile	Outcome
PBL	Mostly present	+ often	Extranodal sites mostly; nodal sites rarely	Frequent (second site after oral cavity)	PBs, IBs (diffuse pattern of growth)	Plasmablast (blastic B cell switched to the PC gene programme)	+ in HIV+ pts and PT pts	-	Monoclonal (IG gene rearrangement); *MYC* translocation (50% of cases)	Poor
PEL	Mostly present	+ (- in elderly and EBV-negative cases)	Classic PEL: serous cavities. EC-PEL: nodal sites mostly; extranodal sites rarely	Rare (EC-PEL)	PBs, IBs (diffuse pattern of growth in EC-PEL; in fluids in classic PEL)	Post-GC cell with PB differentiation	+ (- in elderly HIV-negative pts)	+	Monoclonal (IG genes hypermutated)	Poor
BL	Present in the immunodef associated variant	+ in the immunodef associated v.	Extranodal sites mostly; nodal sites in the Immunodef associated v.	Frequent	Medium-sized cells (diffuse monotonous pattern of growth)	GC B cell	>95% endemic v.; 20–30% sporadic v.; 25–40% immunodef v.	-	Monoclonal (IG gene rearrangement); *MYC* translocation (90% of cases)	Highly aggressive, but potentially curable
EBVMCU	Mostly present (often iatrogenic)	+ rarely	Extranodal sites: oropharynx (most frequent), skin, GIT	Infrequent	Polymorphic infiltrate (HL-like) or monomorphic infiltrate (DLBCL-like)	EBV-transformed post-GC B-cell	+	-	IG or TCR genes clonality in 40% of cases	Mostly good (often spontaneous regression or upon removal of IS cause)
cHL	Rarely present (frequent in MCCHL and LDCHL)	+ rarely (frequent in MCCHL and LDCHL)	Mediastinum and nodal sites	Very rare	Polymorphic infiltrate with HL cells and RSCs	GC B-cell	+ (75% MCCHL; 10–25% NSCHL)	-	IG gene clonality may be present	Curable in 80% of cases
EBV+ DLBCL, NOS	May be present (in the intestinal form)	-	Nodal (frequent in young) and extranodal sites (elderly)	Frequent	Polymorphic or monomorphic pattern of growth	Mature B-cell transformed by EBV	+	-	Monoclonal (IG gene rearrangement)	Poor in elderly patients and intestinal form
HHV8+ GLPD	Absent	- (rarely +)	Nodal sites (mostly single lymph nodes)	No	PBs/IBs (single or in tiny clusters in GC)	GC B-cell	+	+	Polyclonal or oligoclonal (rarely monoclonal)	Mostly good
LYG	Mostly present	- (rarely +)	Extranodal sites (lungs in >90%)	Rare	Polymorphic infiltrate with a variable number of EBV-positive B cells	Mature B-cell transformed by EBV	+	-	Monoclonal (IG gene rearrangement often identified in grade 2 and 3)	Variable from good to poor depending on grading
CAEBV	Absent	-	Extranodal, nodal sites and BM	May be involved	Small or medium-sized lymphocytes with mild or no atypia	CD4+ T cell, NK cells, cytotoxic CD8+ T cell and rarely gamma delta T cells	+	-	TCR clonality often; rarely oligoclonality or polyclonality	Variable from indolent to rapidly fatal (worse outcome in T-cell type and adult-onset)

ENKTL nasal-type	Rare	-	Extranodal sites (upper aerodigestive tract more common)	Frequent	Polymorphic pattern; angiocentric and angiodestructive growth	Activated NK cells and more rarely cytotoxic T cells	+	-	TCR and IG genes mostly germ line; TCR clonality in 10–40%	Variable
PTLPDs	Present (post-transplant occurrence)	-	Extranodal and nodal sites	Frequent	Non-destructive PTLPDs; Polymorphic PTLD; Monomorphic PTLPDs; cHL PTLD	Variable in relation to the category of PTLPDs	+ often	-	Variable depending on the category	Variable depending on the category
Systemic EBV+ T-cell lymphoma of childhood	Absent	-	Liver, spleen, lymph nodes, BM, skin and lungs	Absent	Lymphocytes usually with mild atypia (rare cases with pleomorphic cells)	Cytotoxic CD8+ T cell or activated CD4+ T cell	+	-	Monoclonal (TCR gene rearrangement)	Poor (fulminant course usually complicated by HLH)
HV-like LPD	Absent	-	Skin	Absent	Dermal and subcutaneous lymphoid infiltrate mostly with mild atypia and angiodestructive features; intraepidermal spongiosis	Skin-homing cytotoxic T cell or NK cell	+	-	Monoclonal (TCR gene rearrangement in cases of T cell origin)	Indolent for years (recurrent skin lesions) before systemic involvement; high risk of systemic lymphoma
SMBA	Absent	-	Skin	Absent	Dermal and subcutaneous polymorphic infiltrate, angiodestructive features; often epidermal necrosis	Mature activated NK cell	+	-	Rarely monoclonal TCR gene rearrangement	Long course with risk of HLH, aggressive NK-cell leukemia or NK/T cell lymphoma
Aggressive NK-cell leukemia	Absent	-	BM, PB, liver and spleen, but any organ may be involved	Rare	Often monotonous cells with round nuclei	Activated NK cell	+ mostly	-	TCR genes germline	Fulminant course with HLH, multiorgan failure and coagulopathy
AITL	Absent	-	Nodal sites mostly (spleen, liver, BM and skin often)	Rare	Small to medium-sized monotonous cells with clear cytoplasm within inflammatory background; often HEVs and FDCs hyperplasia	CD4+ TFH cell	EBV + B cells in 80–95% of cases	-	Monoclonal (TCR gene rearrangement in 70–90% of cases)	Poor

PTCL, NOS	Absent	-	Nodal sites mostly, but any site can be affected including extranodal sites	Common	Monomorphic or pleomorphic cells within inflammatory background	Activated mature T cell of CD4+ central memory type	EBV + (more often in background B cells and rarely in neoplastic cells)	-	Monoclonal (TCR gene rearrangement)	Poor
FTCL	Absent	-	Nodal sites mostly	Rare	Medium-sized monotonous cells; follicular pattern or PTGC-like pattern	TFH	EBV+ B cells	-	Monoclonal (TCR gene rearrangement	Poor
CI-DLBCL	Absent	-	Body cavities (often thoracic cavity); mass-forming lesion in longstanding CI	Absent	Sheets of large cells (IBs or CBs)	EBV-transformed post-GC B cell	+	-	Monoclonal (IG genes rearranged and hypermutated)	Poor
FA-DLBCL	Absent	-	Developing within pseudocysts, fibrin thrombi, cardiac myxoma, prosthesis, hematomas; <u>not</u> mass-forming	Absent	Single or tiny clusters of large cells within fibrin	EBV-transformed post-GC B cell	+	-	Monoclonal (IG genes rearranged and hypermutated)	Favorable even with surgery alone (mostly)
Lymphopr. diseases associated with PIDs	Present (PIDs)	-	Extranodal sites mostly (GIT, lungs, CNS)	Frequent	Reactive hyperplasia; polymorphous lymphoid infiltrate; frank lymphoma	B-cell origin in most lymphomas	+	-	Polyclonal, oligoclonal or monoclonal	Variable, depending on type of PIDs (CVID may be indolent; ALPS often self-limited ;most LPDs aggressive)

AITL: angioimmunoblastic T-cell lymphoma; ALPS: autoimmune lymphoproliferative syndrome; BL: Burkitt lymphoma; BM: bone marrow; CAEBV: chronic active EBV infection; CBs: centroblasts; cHL: classic Hodgkin lymphoma; CI: chronic inflammation; CI-DLBCL: diffuse large B-cell lymphoma associated with chronic inflammation; CNS: central nervous system; CVID: common variable immunodeficiency; DLBCL: diffuse large B-cell lymphoma; EBER: in situ hybridization for EBV-encoded RNA; EBV: Epstein-Barr virus; EBV+ DLBCL, NOS: EBV-positive, diffuse large B-cell lymphoma, not otherwise specified; EBVMCU: EBV-positive mucocutaneous ulcer; EC-PEL: extra-cavitary primary effusion lymphoma; ENKTL, nasal type: extranodal NK/T-cell lymphoma; FA-DLBCL: fibrin-associated diffuse large B-cell lymphoma; FDCs: follicular dendritic cells; FTCL: follicular T-cell lymphoma; GC: germinal centre; GIT: gastrointestinal tract; HHV8: Human herpes virus 8; HHV8+ GLPD: HHV8-positive germinotropic lymphoproliferative disorder; HEVs: high endothelial venules; HIV: human immunodeficiency virus; HL: Hodgkin lymphoma; HLH: hemophagocytic lymphohystiocytosis; HV-like LPD: Hydroa vacciniforme-like lymphoproliferative disorder; IBs: immunoblasts; Immunodef: immunodeficiency; IG immunoglobulin; IS: immunosuppression; LDCHL: lymphocyte-depleted classic Hodgkin lymphoma; LPDs: lymphoproliferative disorders; LYG: lymphomatoid granulomatosis; Lymphopr: lymphoproliferative; MCCHL: mixed cellularity classic Hodgkin lymphoma; NSCHL: nodular sclerosis classic Hodgkin lymphoma; PBL: plasmablastic lymphoma; PB: peripheral blood; PBs: plasmablasts; PC: plasma cell; PEL: primary effusion lymphoma; Post-GC: post-germinal centre; PT: post-transplant; PTGC: progressively transformed germinal centre; PTLDs: post-transplant lymphoproliferative disorders; pts: patients; RSCs: Reed Sternberg cells; SMBA: severe mosquito bite allergy; TCR: T-cell receptor; v: variant; TFH: T-follicular helper; **Note**: PBL, EC-PEL and BL are discussed in the current part 2 of the review.
